# Viral exacerbation of chronic airway inflammation in BALB/c mice sensitized with house dust mite (HDM): a role for corticosteroids?

**DOI:** 10.1186/1476-9255-10-S1-P41

**Published:** 2013-08-14

**Authors:** Edward G Barrett, Al P Senft, Jennifer Nicodem, Dylan Armes, Aaron Gaskins, Ginevra Giannini

**Affiliations:** 1 Lovelace Respiratory Research Institute, Albuquerque, NM, 87108, USA

## Background

Respiratory viral infections are commonly associated with the exacerbation of asthma in humans. The development of more predictive animal models that incorporate an exacerbation component are needed to better define translational biomarkers and therapeutic targets.

## Materials and methods

Male BALB/c mice were sensitized to purified house dust mite extract (HDM; 25 µg) without exogenous adjuvant 5 days/week for 5 weeks (day 0-32). On day 21 designated groups were treated with fluticasone propionate (F; 10ug/animal, BID) until the end of the study. On day 35, designated groups were instilled with influenza (IFZ; A/HKx31[H3N2]; 10^4^ PFU). On day 39 (4 days pi) and 49 (14 days pi), groups were evaluated for airway hyperreactivity (AHR), lung inflammation (BAL), viral titer, and histopathology.

## Results

HDM challenge led to an increase in BAL cells (eosinophils, neutrophils, macrophages, and lymphocytes) at day 39 that resolved by study day 49. IFZ only led to a significant increase in BAL cells (neutrophils, macrophages, and lymphocytes) at day 39 that also resolved by study day 49. HDM+IFZ did not lead to a further increase in BAL cells. Fluticasone treatment attenuated the HDM only response, however, BAL cells were significantly increased in the F+HDM+IFZ group (770% ↑ in neutrophils, 310% ↑ in eosinophils). HDM and IFZ alone led to a significant increase in AHR (IFZ day 39 > HDM 39 = HDM or IFZ day 49) that remained elevated at day 49. AHR in HDM+IFZ animals was similar to IFZ alone and remained elevated at day 49 (= HDM or IFZ day 49). Fluticasone treatment in HDM animals had no effect on AHR at day 39 but was effective if continued to day 49 and reduced AHR to nonsensitzed control levels. In contrast, AHR was further increased in F+HDM+IFZ animals (270% ↑ above HDM+IFZ). None of the F+HDM+IFZ mice survived past day 42.

## Conclusions

Viral infection with influenza on a background of established chronic airway inflammation did not induce an exacerbation phenotype, as measured by inflammatory cell counts and AHR. However, in the presence of infection the efficacy of a steroid on the allergic airway response was reversed and led to a significant exacerbation.

